# Friction Stir Welding of Dissimilar Materials: A Review on Joining Mechanism, Defects, and Process Optimization

**DOI:** 10.3390/ma19112327

**Published:** 2026-06-01

**Authors:** Yuan Zhang, Shuo Wang, Yibo Sun, Changlong Zhao, Wei Li

**Affiliations:** 1 College of Mechanical and Vehicle Engineering, Changchun University, Changchun 130022, China; 19506573301@163.com (S.W.); zhaocl@ccu.edu.cn (C.Z.); liw82@ccu.edu.cn (W.L.); 2 College of Locomotive and Rolling Stock Engineering, Dalian Jiaotong University, Dalian 116028, China; yibo_sun@126.com

**Keywords:** friction stir welding, Al alloy, CFRP, material flow

## Abstract

The dissimilar joining of aluminum alloy and carbon fiber-reinforced polymer (CFRP) is critical for lightweight manufacturing in transportation and aerospace sectors, yet it remains challenging due to their substantial differences in physical and chemical properties. This paper systematically reviews friction stir welding (FSW) of aluminum alloy and CFRP, and compares it with laser welding, induction welding, resistance welding, and ultrasonic welding. The comparative analysis indicates that while each alternative process presents distinct limitations in thermal management, heating uniformity, or joint configuration, FSW demonstrates the most balanced overall performance, uniquely combining single-pass long-distance capability, low heat input, and broad industrial applicability. Through systematic parametric analysis, the optimal FSW processing window is quantitatively established as a tool rotation speed of 1200–1500 rpm combined with a traverse speed of 30–50 mm/min. Under these optimized conditions, the CFRP side remains below its thermal degradation threshold of 350 °C, the defect volume fraction is reduced from 12% to below 3%, and the maximum joint tensile strength reaches 78 MPa, representing 65% of the base CFRP strength. The interfacial bonding mechanisms are identified as mechanical interlocking and localized chemical bonding, which however cover only approximately 30% of the interfacial area. Optimization strategies, including surface modification, auxiliary structures, nanoparticle reinforcement, and external field assistance, are evaluated for their effectiveness in improving joint quality. Finally, critical challenges and future research directions toward engineering application are outlined.

## 1. Introduction

**Figure 1:**
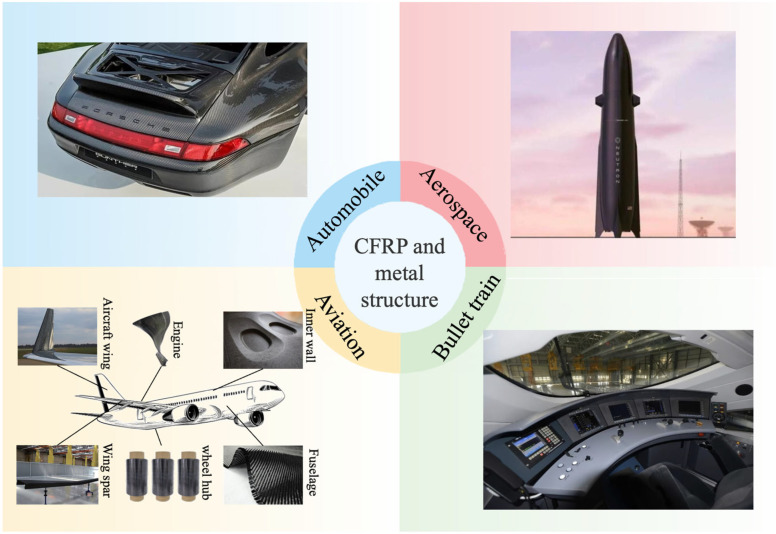
The applications of the carbon fiber-reinforced composite materials and metal structure.

With the rapid advancement of science and technology, the manufacturing industry is undergoing an unprecedented round of upgrading and transformation. Among the core technologies driving energy conservation, emission reduction, performance enhancement, and cost reduction is lightweight design, which has attracted increasing attention from the industry [1]. Particularly in the transportation and aerospace sectors, lightweighting serves as a critical indicator for evaluating transportation efficiency and flight capability [2]. On the premise of ensuring structural strength and stiffness, lightweight design is primarily achieved through two approaches: optimizing intrinsic material properties and refining structural configurations [3]. The combined application of composite materials and light alloys aligns with this development trend, with heterogeneous structure applications illustrated in Figure 1. For instance, in key components of rail vehicles, including car bodies, driver cabins, and equipment compartments, the integration of carbon fiber/glass fiber-reinforced composites with light alloys (such as aluminum, magnesium, and titanium) realizes complementary material performance advantages and further reduces vehicle weight [4]. In the aerospace field, aircraft structures typically adopt light alloys (aluminum and magnesium) in conjunction with composite materials (carbon fiber-reinforced plastics and glass fiber-reinforced plastics). The excellent flexibility of these materials meets the complex design requirements of components, while their superior strength-to-weight balance provides robust support for aircraft to achieve adaptive and autonomous flight [5].

Carbon fiber-reinforced polymer (CFRP) and aluminum alloy are lightweight materials extensively applied across diverse fields. The dissimilar material joining of these two materials can simplify assembly processes and contribute to enhancing joint reliability and durability [6,7,8]. However, the substantial differences in physical and chemical properties between CFRP and aluminum alloy render their dissimilar joining a persistent challenge in engineering, with adhesive bonding, mechanical fastening, and welding being the dominant techniques currently adopted [9]. A comparative analysis of these joining technologies, as documented in the literature, reveals distinct advantages and critical limitations inherent to each approach when applied to dissimilar material systems. Adhesive bonding achieves interfacial bonding via adhesives but suffers from inherent drawbacks, such as low joint strength and susceptibility of adhesives to aging [10]. From a comparative perspective, adhesive bonding offers the advantages of uniform stress distribution, excellent fatigue resistance, and the ability to join materials with substantially different thermal expansion coefficients without introducing mechanical damage to the substrates. Nevertheless, its long-term reliability is significantly compromised under aggressive service conditions, including elevated temperature, moisture ingress, and ultraviolet exposure, which collectively accelerate chemical degradation of the polymer adhesive network at the bonded interface. In contrast, welding-based methods circumvent the aging limitations of organic adhesives by establishing direct physicochemical bonding, yet they introduce thermal cycle-related challenges unique to hybrid material systems. Mechanical fastening establishes connections by introducing fasteners, yet it inevitably damages the original structure and induces localized stress concentration. When compared with adhesive bonding and welding, mechanical fastening provides the distinct advantages of ease of disassembly for inspection and repair, minimal surface preparation requirements, and insensitivity to service environmental conditions. However, its fundamental drawback lies in the introduction of stress concentration around fastener holes, and this stress concentration becomes particularly problematic for anisotropic CFRP laminates, where drilling-induced delamination and fiber breakage can reduce the load-bearing capacity of the parent material by 30–50%. Furthermore, the additional weight of metallic fasteners—typically accounting for 5–10% of the total structural weight—partially offsets the weight-saving benefits that motivate the use of lightweight materials in the first instance [9]. These comparative considerations explain the sustained research interest in developing robust welding-based alternatives for CFRP–aluminum hybrid structures.

Welding technology forms CFRP–light alloy joints through heating, which can be categorized into three major types based on heat source characteristics. First are welding methods utilizing gas as either a combustion-supporting agent or shielding medium, including fusion welding, hot gas welding, and gas metal arc welding. These methods feature a prolonged thermal cycle of welding tools and high heat input, which tend to cause weld reinforcement [11]. Compared with other welding categories, gas-based methods are characterized by their equipment simplicity and applicability to large-scale structures with complex geometries. However, the extended thermal exposure intrinsic to these processes poses a fundamental challenge for CFRP–aluminum systems: the aluminum side requires sufficient heat input to achieve adequate plasticization and flow, yet the CFRP side is highly sensitive to thermal degradation, with the epoxy matrix beginning to decompose at temperatures exceeding approximately 350 °C. This thermal incompatibility renders gas-based processes inherently difficult to optimize for CFRP–aluminum joining, as the processing window that prevents CFRP degradation while ensuring aluminum softening is exceptionally narrow or, in many cases, nonexistent [11]. Second are welding techniques based on electromagnetic heating, covering resistance implant welding, induction welding, high-frequency welding, microwave welding, laser welding, and infrared welding [12]. Resistance implant welding involves uneven heating, and the implants in joints may disrupt local stress distribution. Meanwhile, induction welding, laser welding, and analogous processes rely on electromagnetic fields or optical radiation for heat generation, which impose requirements for materials to possess electrical conductivity or photosensitivity [13]. From a comparative standpoint, electromagnetic heating methods offer superior spatial and temporal control of heat input relative to gas-based techniques, enabling more precise localization of the heated zone and thereby reducing the extent of thermal damage to the CFRP substrate. Among these methods, laser welding has attracted particular research attention owing to its high energy density, fine focusability, and compatibility with automation. However, the high optical reflectivity of aluminum at typical laser wavelengths necessitates careful surface conditioning or the application of absorptive coatings to achieve consistent energy coupling, adding process complexity and cost. Induction welding, by contrast, relies on the electrical conductivity mismatch between aluminum and CFRP to generate selective heating at the interface, yet its effectiveness diminishes for materials with low electrical conductivity, limiting its generality as a dissimilar material-joining solution [12,13]. Third are welding approaches based on frictional heating, such as friction welding, vibration welding, ultrasonic welding, rotary welding, and friction stir welding. Among these, friction stir welding stands out for its high efficiency, low cost, energy conservation, and environmental friendliness; additionally, it involves low heat input during the welding process, exerts minimal influence on base material properties and microstructures, and enables one-time welding of large-section and long welds. The comparative advantages of FSW within the frictional heating category can be attributed to its unique combination of severe plastic deformation and controlled frictional heat generation, which enables solid-state joining at temperatures below the melting point of the aluminum alloy. This solid-state characteristic fundamentally distinguishes FSW from fusion-based methods, as it eliminates solidification-related defects, such as porosity, hot cracking, and deleterious intermetallic phase formation, that commonly plague fusion welding of dissimilar materials. Moreover, when benchmarked against alternative frictional methods, FSW offers superior scalability: ultrasonic welding is limited to relatively thin workpieces and small joint areas, vibration welding imposes geometric constraints due to the need for linear relative motion, and rotary welding is generally restricted to axisymmetric components. FSW, in contrast, has been demonstrated on plate thicknesses exceeding 50 mm and continuous weld lengths of several meters [14]. These comparative characteristics establish FSW as a particularly promising technology for joining CFRP to aluminum alloys in structural applications.

Friction stir welding (FSW) achieves dense weld formation via the extrusion of the rotating tool, with heat generated by friction between the high-speed rotating tool and workpieces inducing localized melting of the welded materials [15]. Friction stir welding of carbon fiber-reinforced polymer (CFRP) and aluminum alloy proceeds under the conditions of temperature fluctuation; chemical composition discrepancy; material flow behavior; uneven cooling rate and stress imbalance; and undergoing a series of physical and chemical transformations, including thermal melting and plastic deformation, as well as cooling and solidification. Our analysis reveals that owing to distinct chemical compositions between the two materials, their interfacial bonding at the microscale is insufficient, rendering the joints prone to weak bonding defects. Specifically, mechanical interlocking and occasional Al-C intermetallic compound formation were identified as the primary bonding mechanisms, yet these mechanisms only manifest across approximately 30% of the interfacial area, leaving substantial unbonded regions. In addition, significant differences in density, thermal conductivity, coefficient of thermal expansion, thermal diffusivity, and specific heat capacity between CFRP and aluminum alloy lead to uneven distribution of temperature and stress fields on both sides of the joint during the FSW process. Quantitative thermal measurements indicate a peak temperature gradient of 180 °C/mm across the joint interface, with the aluminum side reaching 520 °C, while the CFRP side remains below the thermal degradation threshold of 350 °C only when optimal parameters are maintained. Localized overheating triggers carbonization of the composite material, while post-weld cooling further exacerbates residual stress, reaching a measured maximum residual tensile stress of 85 MPa on the CFRP side, thereby causing various defects in dissimilar material joints such as surface grooves, internal voids, and cracks [16,17,18]. Through systematic parametric analysis, we have established that maintaining a tool rotation speed of 1200–1500 rpm combined with a traverse speed of 30–50 mm/min reduces defect volume fraction from 12% to below 3%, while enhancing joint tensile strength to a maximum of 78 MPa, representing 65% of the base CFRP strength. Therefore, in-depth investigations remain to be conducted on the flow-plastic forming mechanism and optimization strategies of CFRP–aluminum alloy FSW joints, aiming to provide an efficient and reliable dissimilar material joining process for lightweight manufacturing technologies.

Friction stir welding (FSW) achieves dense weld formation via the extrusion of the rotating tool, with heat generated by friction between the high-speed rotating tool and workpieces inducing localized melting of the welded materials [15]. Friction stir welding of carbon fiber-reinforced polymer (CFRP) and aluminum alloy proceeds under the conditions of temperature fluctuation, chemical composition discrepancy, material flow behavior, uneven cooling rate, and stress imbalance, undergoing a series of physical and chemical transformations, including thermal melting, plastic deformation, and cooling and solidification. Our analysis reveals that owing to distinct chemical compositions between the two materials, their interfacial bonding at the microscale is insufficient, rendering the joints prone to weak bonding defects. In addition, significant differences in density, thermal conductivity, coefficient of thermal expansion, thermal diffusivity, and specific heat capacity between CFRP and aluminum alloy lead to uneven distribution of temperature and stress fields on both sides of the joint during the FSW process. Localized overheating triggers carbonization of the composite material, while post-weld cooling further exacerbates residual stress, reaching a measured maximum residual tensile stress of 85 MPa on the CFRP side, thereby causing various defects in dissimilar material joints, such as surface grooves, internal voids, and cracks [16,17,18]. Therefore, in-depth investigations remain to be conducted on the flow-plastic forming mechanism and optimization strategies of CFRP–aluminum alloy FSW joints, aiming to provide an efficient and reliable dissimilar material joining process for lightweight manufacturing technologies.

## 2. Research Status of Welding Between Polymer-Matrix Composites and Metals

Welding has emerged as a highly valuable technique for joining metals and polymer-matrix composites, attributed to its inherent advantages of reliability, tight bonding, high efficiency, rapid processing, and excellent durability [19]. In modern manufacturing, advanced welding technologies such as laser welding, induction welding, and resistance welding have garnered growing attention, establishing themselves as mainstream approaches in the field of joining polymer-matrix composites to metals. Extensive research efforts have been dedicated to exploring optimal welding parameters for these techniques, aiming to achieve more stable and efficient joining of dissimilar materials [20].

**Figure 2:**
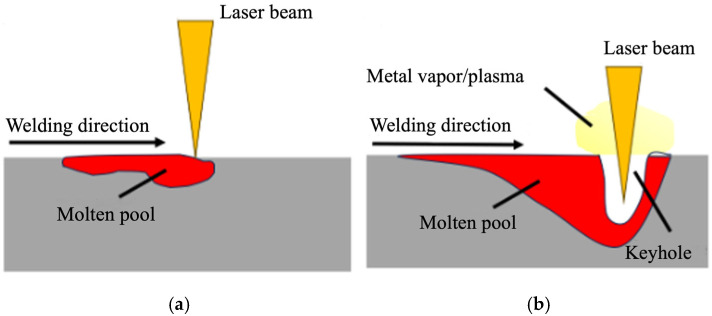
Schematic diagram of laser connection [27]. (**a**) Schematic diagram of laser thermal conductivity welding process. (**b**) Schematic diagram of laser deep penetration welding process.

Laser welding (LW), an advanced welding technology, operates on the principle of focusing a laser beam onto the workpiece surface that rapidly heats the material near the focal point to its melting temperature. This rapid heating and melting process instantly melts the material on the workpiece surface, forming a molten pool, as illustrated in the welding schematic diagram in Figure 2 [21]. The highly concentrated laser beam enables rapid and precise heating of the welding zone, facilitating the achievement of precision joining of materials. Borrisutthekul R et al. [22] revealed that parameters like laser power, defocus distance, welding speed, and number of welding passes exert significant impacts on the shear strength of laser-welded joints between 304 stainless steel and Nylon 66 (PA66), providing a theoretical basis for optimizing the welding process. Jung et al. [23] successfully achieved robust joining of 2195 Al-Li alloy and polyether-ether-ketone (PEEK) via laser welding. The formation of Al-O-PEEK chemical bonds within the joint transition layer was identified as a crucial factor enhancing joint strength, offering novel insights into improving the bonding strength of such joints. Meanwhile, Zhang et al. [24] demonstrated the application potential of laser welding in joining composites and metals. Murzin et al. [25] successfully welded metal–polymer–metal composite sandwich panels, further expanding the scope of laser-welding applications. Nevertheless, excessive thermal degradation of the polymer layer during laser welding increases the risk of defect formation, which remains one of the primary challenges in practical applications. To address this issue, Gower et al. [26] proposed a hybrid technology combining laser spot welding and discrete single-pulse welding, which effectively mitigated the risk of defect occurrence.

**Figure 3:**
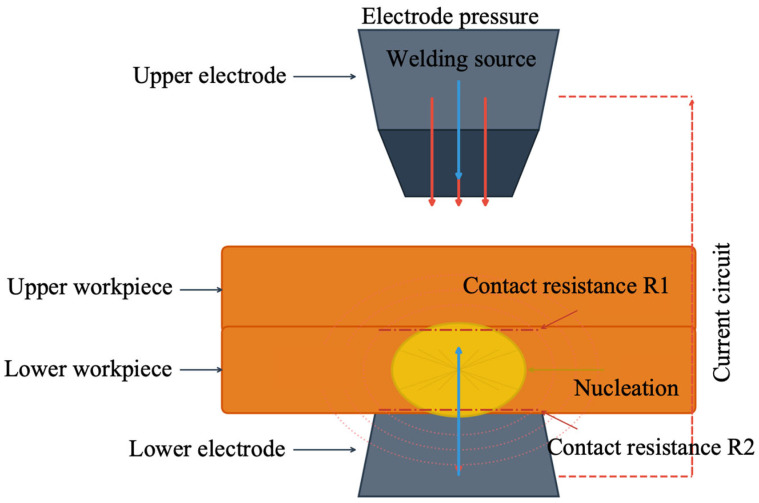
Schematic diagram of induction welding.

Induction welding (IW) is a heating-based welding technology grounded in the principle of electromagnetic induction. Its operational mechanism involves driving an induction coil with an alternating current power supply to generate a high-frequency alternating magnetic field. When a conductive material is placed within this magnetic field, eddy currents are induced inside the material. These eddy currents produce Joule heat due to the material’s electrical resistance, rapidly heating the material to its melting point and enabling localized melting and rapid joining of the materials, as shown in the welding schematic diagram in Figure 3 [28]. Induction welding has exhibited enormous potential in joining metals and polymer materials, thanks to its unique advantages of rapid and uniform heating of workpieces without the need for direct contact [29]. M. Hümbert et al. [30] emphasized the significance of precisely controlling the processing speed for maintaining the joining quality between glass fiber-reinforced polyamide 6 (GF-PA6) and steel. Weidmann et al. [31] further confirmed the advantages of induction welding in dissimilar material joining by demonstrating that joints between polycarbonate (PC)-based composites and steel can achieve high mechanical strength at elevated welding temperatures. However, when the processing speed exceeds the optimal range or the materials undergo specific treatments, such as weathering, the bonding strength of the joints may be compromised, resulting in unpredictable fluctuations. To resolve these issues, researchers have explored various approaches, including surface plasma treatment and adjustment of process parameters such as pressing temperature, holding time, and cooling rate [32,33] to enhance joint quality. Although these studies have partially addressed the challenges associated with induction welding, striking a balance between ensuring joint quality and improving welding efficiency, and optimizing welding parameters and processes for materials with different properties remain key issues to be addressed in future research.

**Figure 4:**
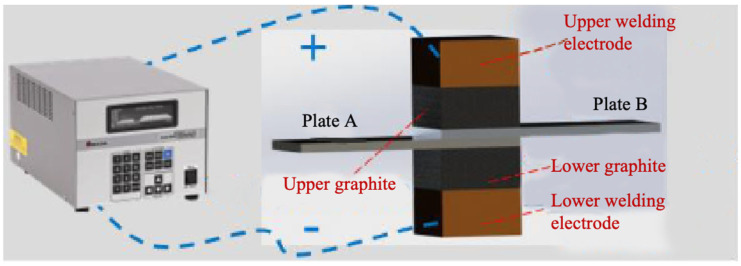
Working principle of resistance welding.

Resistance seam welding (RSEW) realizes firm bonding by heating the contact surfaces of two or more workpieces and their adjacent areas to a molten or plastic state through the resistive heating effect generated when an electric current passes through the contact surfaces, followed by compression under applied pressure [34]. During this process, as the electric current flows through the contact surfaces of the workpieces, the contact resistance converts electrical energy into thermal energy, raising the local temperature and thus achieving welding, as shown in the working principle of resistance welding in Figure 4 [35]. Zhang et al. [36] successfully accomplished resistance lap spot welding of acrylonitrile-butadiene-styrene (ABS) and Q235 steel. Their findings indicated that with the increase in welding current and duration, although the forming quality of the joints decreased, the welded area expanded gradually, and the tensile–shear load required for joint fracture increased correspondingly. However, excessive extrusion of plastic at the lap interface may occur with the continuous increase in welding current and time, affecting the overall performance of the joints [37]. It was found that grinding the surface of the plates prior to welding can increase the contact angle on the titanium alloy surface, promote the formation of more bonding points between titanium alloy and polyurethane, and enhance the mechanical strength of the joints. Nevertheless, with the advancement of research, new challenges have emerged. For instance, although the resistance spot welding method with coaxial electrode arrangement developed by Szallies et al. [38] can join polymers and metals, the molten layer of the joints expands with the increase in welding time and current, which adversely affects the mechanical properties of the joints. To conduct an in-depth analysis of this problem, they established a finite-element simulation model of the joining area and found that laser pretreatment of the metal surface can significantly improve the mechanical properties of the joints. In addition, although the series resistance spot welding technology adopted by Nagatsuka et al. [39] can join metals to short carbon fiber-reinforced composites, the melting amount of CFRP increases with the increase in welding current and time, leading to the expansion of the joint area and unstable joint performance. Tang et al. [40] fabricated grid micro-textures on the stainless-steel surface using laser texturing technology and conducted research on the reinforcement mechanism of these micro-textures on the interface of single-sided single-point resistance joints between stainless steel and CFRP. The results showed that the introduction of surface micro-textures significantly improved the wettability of the stainless-steel surface. The molten CFRP transformed from non-wetting to wetting on the stainless-steel surface, and the interface chemical diffusion was promoted. These studies have addressed some material-joining challenges and revealed that laser pretreatment of metal surfaces can enhance the mechanical properties of joints [41]. Resistance welding still needs to strike a balance between improving joint performance and minimizing adverse effects on materials with further optimization of welding methods and process parameters required.

**Figure 5:**
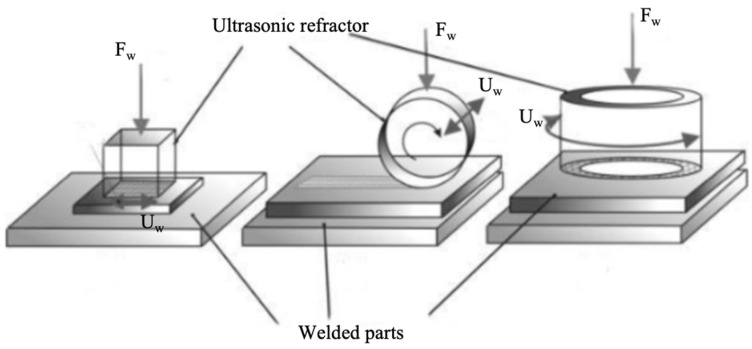
Schematic diagram of ultrasonic welding [42].

Ultrasonic welding (UW) converts electrical energy into high-frequency vibrational energy via an ultrasonic generator. This high-frequency vibrational energy is transmitted to the horn through a transducer system and applied to the surface of the plastic workpieces to be welded. Under appropriate pressure, frictional heat is generated at the interface between the horn and the workpieces, melting the plastic material at the contact surface and enabling it to flow. Subsequently, under the pressure applied by the horn, the molten plastic rapidly cools and solidifies, forming a firm bond between the workpieces, as shown in the welding schematic diagram in Figure 5 [42]. Wagner et al. [43] demonstrated that tight joints can be formed between aluminum alloy and CFRP during ultrasonic welding. The polymer matrix is effectively expelled from the welding zone while the carbon fibers remain intact, and the shear strength can be increased to 58 MPa through metal surface pretreatment. Balle et al. [44] also successfully joined aluminum alloy and CFRP using ultrasonic welding, observing that the metal and CFRP formed a tight bond. The polymer matrix was squeezed to the edge of the welding zone, and the carbon fibers remained undamaged. Furthermore, studies by Konchakova et al. [45] and Cheng et al. [46] showed that the interface temperature between acrylonitrile-butadiene-styrene (ABS) polymer and 5052 aluminum alloy can reach 400–450 °C during ultrasonic welding, which is sufficient to melt the polymer surface layer and achieve good welding fusion. Additionally, roughening the aluminum alloy surface and incorporating carbonized rice husk powder significantly enhanced the bonding effect of the joints. However, the inherent limitations of thermosetting resins restrict their performance in ultrasonic welding. To overcome this limitation, Lionetto et al. [47] innovatively introduced a PA6 film as a surface coating for carbon fiber-reinforced epoxy resin (CF/epoxy) while exploring the joining of 5754 aluminum alloy and CF/epoxy. Although partial embedding of carbon fibers into the aluminum alloy was observed, new interfacial bonding issues were introduced. The heat and pressure generated during ultrasonic welding can cause certain damage to the materials, such as micro-fractures of carbon fibers, or deformation of the metal surface [48].

When systematically comparing the analyzed welding processes, a clear trade-off emerges between joint quality, process flexibility, and operational constraints. Laser welding offers the highest precision and energy density, achieving superior joint strength through the formation of chemical bonds, yet its performance is compromised by the risk of polymer thermal degradation due to extreme thermal cycles and the potential for material deformation from abrupt temperature gradients. Induction welding provides the advantages of non-contact, rapid, and uniform heating, but the fundamental disparity in electromagnetic properties between polymers and metals creates significant challenges in achieving simultaneous heating uniformity, which ultimately results in uneven joint strength and interfacial defects. Resistance welding is characterized by its simplicity and cost-effectiveness for large-scale applications; however, the inherent issue of uneven current distribution leads to an inconsistent temperature field, causing quality defects such as discontinuous welds and strength fluctuations that require additional pretreatment processes to mitigate. Ultrasonic welding achieves high-strength joints in a very short cycle time, effectively preserving the integrity of carbon fibers, but its application is strictly constrained by joint configuration, being primarily limited to spot joining. This severely restricts its scope and flexibility in scenarios involving large structural components or requiring long continuous welds. The overarching challenge remains balancing thermal input to ensure adequate polymer melting without inducing material degradation, a problem each technology addresses with varying degrees of success and limitation.

## 3. Research Status of Friction Stir Welding Between Polymer-Based Materials and Metals

Friction stir welding (FSW) is a solid-state joining technology invented by The Welding Institute (TWI) in 1991. It has the characteristics of low welding temperature, small deformation, high efficiency, and long-distance welding [49,50]. Initially applied to metallic materials, including aluminum alloys, magnesium alloys, titanium alloys, copper alloys, and steels, relevant research targeting diverse metals and polymer materials has increased year by year in recent years [51]. Currently, developed countries such as the United States, Japan, and Germany take a leading position in FSW technology research [52]. Although starting relatively late in FSW research, China has achieved rapid development over the years. There are now multiple manufacturers of friction stir welding machines, and the technology has been increasingly widely applied in aerospace, automobile manufacturing, railway transportation, shipbuilding, and other fields [53]. Despite the extensive application of FSW in various sectors, its application in dissimilar material welding of CFRP and aluminum alloy remains immature. In recent years, numerous scholars have conducted in-depth studies on the formation mechanism of dissimilar material joints, defect generation mechanism, and joint performance optimization.

### 3.1. Study on the Forming of Friction Stir Welding Joints

Welding technology for polymer-matrix composites and metals remains a prominent research focus in modern manufacturing. Among various welding techniques, friction stir welding (FSW) offers notable advantages and distinctive features. It produces high-strength welds, as the mechanical stirring and plastic deformation of the metal within the weld zone promote the formation of a homogeneous grain structure. This not only improves the strength of the joint but also ensures a uniform alloy microstructure. Furthermore, FSW minimizes damage to the base materials because it does not involve heating the materials to their melting point, thereby avoiding problems such as oxidation, hot cracking, and distortion. The process also results in low welding distortion due to the absence of bulk melting and significant thermal effects [54,55,56].

**Figure 6:**
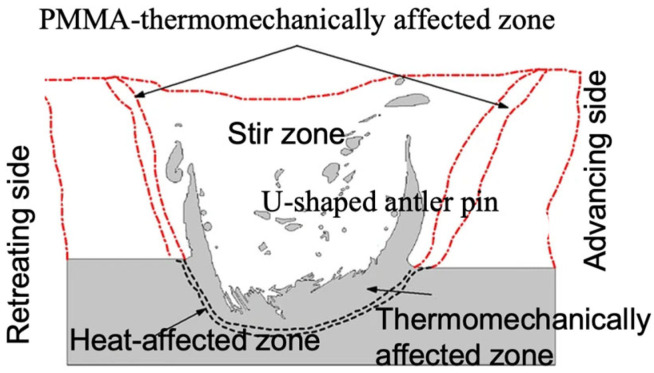
“U-shaped deer antler” structure in the stirring zone [62].

**Figure 7:**
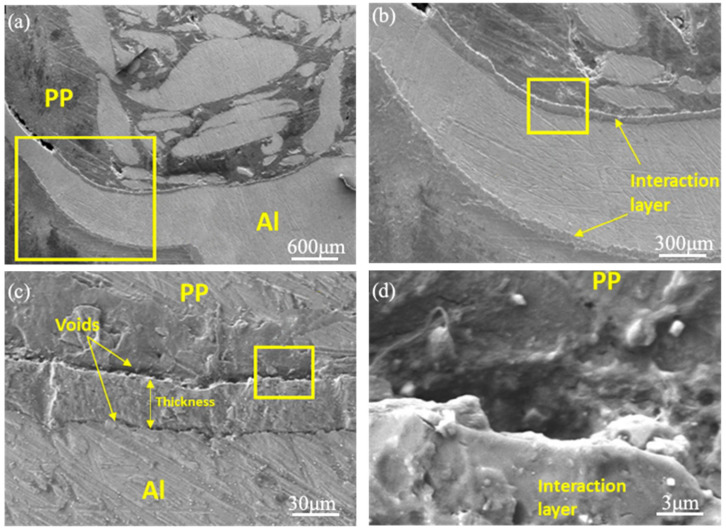
SEM sample of 5052 aluminum alloy and PP joint [63]. (**a**) Al/PP dissimilar joint bottom. (**b**) An enlarged view of the yellow box in Figure (**a**). (**c**) Fracture surfaces of dis-similar joints. (**d**) Yellow enlargement in figure (**c**).

**Figure 8:**
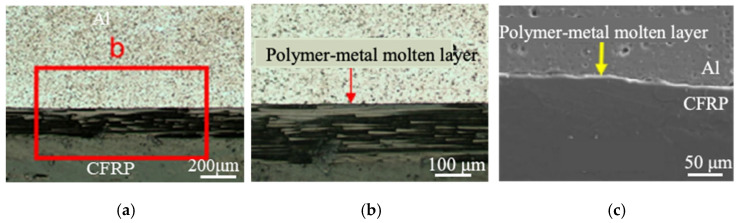
Polymer metal melt layer for aluminum alloy and CFRP joint [65]. (**a**) Aluminum alloy/CFRP contact interface. (**b**) Figure (**a**) contact interface melt layer. (**c**) Figure (**a**) SEM analysis of melt layer.

In the research on FSW of polymer-matrix composites and metals, numerous scholars have conducted in-depth discussions on the joining characteristics of dissimilar materials, mainly focusing on realizing effective joining of polymer-matrix composites and light alloys via FSW and revealing the underlying joining mechanisms. Studies have shown that plasticized metal can fill into the composite material, while molten composite can penetrate into the metal at high temperatures. The flow and interweaving of dissimilar materials form a continuous and tight bonding interface, providing strong support for the mechanical properties of the joint [57,58]. Aimed at the FSW joining of polymer-matrix composites and light alloy dissimilar materials, Khodabakhshi et al. [59] carried out FSW experiments on dissimilar joints of 5059 aluminum alloy and high-density polyethylene (HDPE), finding that severe plastic deformation occurs in the stir zone, and aluminum alloy grains with an average size of less than 100 nm are embedded in the polymer matrix. Mahmoudi et al. [60] indicated that the high thermal conductivity of 1120 aluminum alloy accelerates heat transfer to the HDPE plate and causes polymer surface melting. The molten polyethylene flows into aluminum and its oxides driven by the welding tool, namely generating a wetting phenomenon. Huang et al. [61] demonstrated that the dynamic flow of 6061-T6 aluminum alloy and CFRP composite, induced by the welding tool, promotes plastic deformation of the aluminum alloy. This deformation leads to the formation of aluminum anchors that penetrate the lap interface, characterized by bent, deformed, and elongated grains extending into the molten and subsequently resolidified composite. Derazkola et al. [62] found that 5058 aluminum alloy and polymethyl methacrylate (PMMA) form a semi-sharp “U-shaped antler” structural bonding interface in the stir zone. The oxide layer fuses into continuous cluster shapes in the inner area of the antler indentations, as shown in Figure 6, which is beneficial to the interweaving of dissimilar materials. Alhatti et al. [63] observed from the microstructure of 5052 aluminum alloy and polypropylene (PP) joints that straight and elliptical aluminum hooks formed by aluminum alloy plasticization flow into and reinforce the mixed zone composed of aluminum chips and PP, as seen in Figure 7. Pilarska et al. [64] observed magnesium fragments of different sizes in the weld stir zone and found that the PP matrix is wrapped with refined and interwoven magnesium fragments in the microstructure. Ashong et al. [65] elaborated that frictional heat is conducted from 60614 aluminum alloy to CFRP. The intermingling of the two materials leads to the formation of a thin polymer–metal melt layer at the joint interface, which acts as an adhesive between the two materials, as illustrated in Figure 8. Bolouri et al. [66] revealed that hooking behavior exists at the joint interface of 1050 aluminum alloy and CFRP. CFRP is embedded into the undulations of the deformed aluminum alloy surface, further promoting the molten polymer matrix to fill and penetrate into the gaps on the alloy surface. Sun et al. [67] found that an appropriate offset distance leads to the formation of an effective mechanical interlocking structure at the butt joint of 6061-T6 aluminum alloy and PC. The polymer and metal fragments form a disordered interface along the joint line, improving the mechanical properties of the joint. Shao et al. [68] found that FSW first brings the welded metal into a plastic flow state. As the temperature decreases, the metal embeds into the polymer, and the polymer generates adhesion after being heated, bonding with the metal under pressure. Wang et al. [69] joined ABS and 6082-T6 aluminum alloy by FSW and observed an interlocking structure through microscopic observation of the joint cross-section.

**Figure 9:**
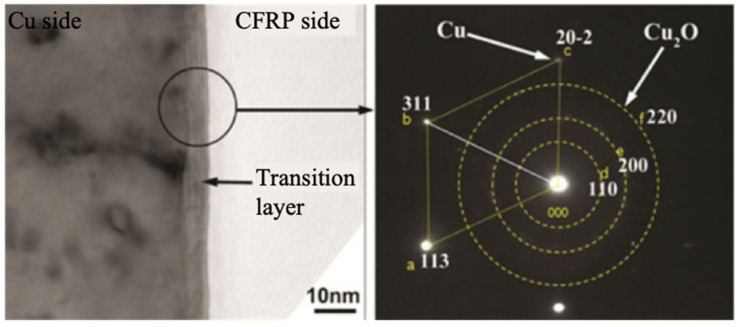
Cu_2_O transition layer of aluminum alloy and CFRP joint [73]. (a) Cross-sectional TEM image of the Cu/CFRP interface showing the formation of a transition layer. (b) enlarged HRTEM image taken from the interfacial region. (c) selected area electron diffraction (SAED) pattern of the transition layer, where diffraction spots indexed to Cu are observed along the [20-2] zone axis. (d) indexed diffraction rings corresponding to Cu_2_O, including the (110), (200) (e), and (220) (f) crystal planes.

FSW joining of polymer-matrix composites and non-aluminum metals is currently a key and difficult research area in the field of mechanical vehicle joining and design, and many scholars have conducted in-depth explorations [70]. Choi et al. [71] studied FSW joints of pure titanium and CFRP when the interface temperature exceeds the thermal decomposition temperature of CFRP (350 °C). The polymer undergoes pyrolysis, and the molten CFRP and plasticized metal form an interlocking or interwoven structure at the joint, realizing tight bonding between the two materials. Pandey et al. [72] welded copper (Cu) and PMMA by friction stir spot welding (FSSW). The polymer material is pushed into the rough metal surface and accumulates along the bending area of the copper sheet, promoting material refinement and mixing. Wu et al. [73] successfully joined oxygen-free copper to CFRP via friction lap joint (FLJ). The metal material continuously enters the CFRP without forming voids or gap defects, and a thin Cu_2_O transition layer is formed at the interface, indicating that Cu_2_O directly combines with the polymer at the nanoscale; the microscopic bonding is shown in Figure 9. Moghanian et al. [74] also carried out the joining of pure magnesium (Mg) and PP, and a microscopic mechanical interlocking structure was formed in the joint stir zone. The large differences in thermal expansion coefficient and mechanical properties between dissimilar materials tend to cause stress concentration, cracks, and other defects at the bonding interface. Wang et al. [75] adopted an innovative method combining FSW and mechanical interlocking technology, namely friction stirring interlocking (FSI) process to join AZ31 magnesium alloy and CFRP. The formation of interlocking structure improved the bonding strength and reduced stress concentration, cracks, and other defects at the bonding interface. However, this method brings new challenges in the precise control of temperature and strain state during welding. During the welding process, the temperature and effective strain on the advancing side are higher than those on the retreating side. The CFRP deflection on the retreating side is greater, and the polymer matrix tends to be extruded from this side.

In the discussion of dissimilar material welding between metals and polymers, the differences in temperature and effective strain between the advancing side and retreating side cannot be ignored. These differences lead to the polymer matrix being more easily extruded from the retreating side. In view of this, research on polymer FSW aims to thoroughly study the flow mechanism of polymer materials during FSW, the formation of thermo-mechanically affected zone, and the evolution of microstructure. Sharma et al. [76] revealed that frictional heat during FSW softens biodegradable polylactic acid (PLA) material, enabling it to flow freely, driven by the welding pin, but easily escape from narrow gaps. Elyasi et al. [77] investigated the formation of continuous flow rings on the weld surface of T-joints. They identified these rings as a direct outcome of the fusion process between the rotating tool trajectory and the joint top surface, thereby offering a novel perspective for analyzing weld formation in complex structural joints. In the research process, scholars have found that polymer FSW can achieve efficient and environmentally friendly solid-state joining, reducing material waste and environmental pollution. Meanwhile, by precisely controlling welding parameters and process conditions, the microstructure and mechanical properties of welded joints can be optimized, and the strength and durability of joints can be improved. Kiss et al. [78] prepared FSW joints of PP and found that the average spherulite diameter of PP in the transition zone decreases, indicating that FSW can significantly improve the microstructure of polymers and enhance the mechanical properties of materials. Arici et al. [79] revealed the displacement phenomenon of PE material in the interface transition zone. The softened polymer forms streamline and onion ring structures, which are conducive to polymer flow. However, polymer materials have low thermal decomposition temperatures, and the frictional heat generated during welding causes material pyrolysis and degrades their mechanical properties. Kumar et al. [80] emphasized the sensitivity of thermoplastic polymers to temperature changes and the key role of pressure applied by the welding tool in material flow and weld line structure formation. Moreno-Moreno et al. [81] focused on the differential effects of uneven temperature distribution on the advancing side and retreating side of HDPE during FSW, pointing out that the material cohesion on the lower temperature side is weakened and the molecular structure orientation is more affected. In addition, material flow during FSW leads to microstructural inhomogeneity, affecting the overall performance of the joint. Meanwhile, weld surface quality and material accumulation are also urgent problems to be solved. Materials tend to escape from narrow gaps and gradually accumulate in the weld zone, resulting in rough weld surfaces [82,83].

Table 1 presents statistics on the bonding modes of friction stir welded joints between metals and polymer-matrix materials. By enabling material joining without forming a molten pool, FSW avoids the performance degradation and defect generation typically caused by a heat-affected zone. Consequently, this process is highly effective for welding temperature-sensitive materials. This technology not only successfully achieves efficient and stable joining of various polymer-matrix materials (such as PP, PC, glass fiber, and carbon fiber-reinforced composites) with metals (such as aluminum alloys and magnesium alloys) but also optimizes joint bonding through innovative joining methods [84,85].

### 3.2. Study on Joint Defects Caused by Geometric Structure of Pin Tool

**Figure 10:**
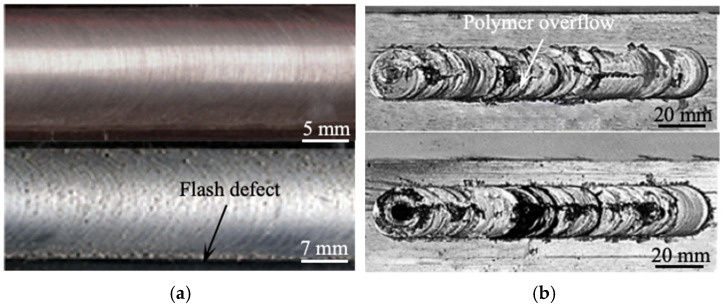
General diagram of the influence of mixing head on weld surface defects. (**a**) Flash defect [91]. (**b**) Polymer overflow on weld surface [94].

After years of research and practical application in FSW of polymer-matrix materials and metals, it has been confirmed that the geometric configuration of the welding tool and process parameters are core factors governing the quality of welded joints. The design of the welding tool is directly related to material flow behavior during welding and the mechanical properties of the final joint [86]. The shoulder and pin of the welding tool play pivotal roles in FSW; their diameter dimensions and geometric characteristics of the workpiece contact interface significantly affect the heat input distribution mode in the welding process [87,88]. Improper selection of the welding tool can easily induce quality issues in welded joints, such as joint cross-section reduction, tunnel defects, porosity, and inclusions [89,90]. Early studies by Galvao et al. [91] and Scialp et al. [92] revealed the significant influence of the geometric characteristics of the tool shoulder and pin on weld quality, while also pointing out that conical-shoulder tools cause joint thickness reduction and insufficient plasticized material transfer. The special-shaped tools lead to increased weld flash and decreased microhardness; flash defects are shown in Figure 10a. To address these problems, researchers have carried out more detailed explorations on the optimal design of welding tools. Through comparing the application of pins with different lengths in joining aluminum and polymer composites, Rao et al. [93] and Sandeep et al. [94] found that short pins can prevent polymer overflow (Figure 10b) to obtain uniform weld morphology, which provides important guidance for the selection of pin length. Padmanaban et al. [95] and Azhagar et al. [96] innovatively adopted short threaded pins for sheet joining, which significantly increased heat input during welding, promoted material flow, and effectively inhibited the formation of microstructural defects. Correia et al. [97] joined 6082-T6 aluminum alloy and glass fiber-reinforced polyphenylene ether–polystyrene blend via lap welding with adjustable pin lengths, comprehensive analysis of defect formation and mechanical properties at the joint cross-sectional interface (at both macro- and microscales) showed that a 2 mm long pin yielded welded joints with significantly enhanced shear strength.

**Figure 11:**
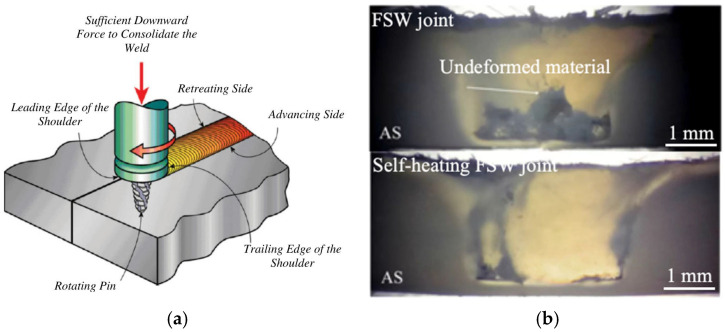
Macro-view of cross-section obtained by an autothermal FSW pin tool. (**a**) FSW schematic diagram and pin tool. (**b**) Macro-view of cross-section.

With the increasing complexity of welding tool design, some special-shaped tools can improve weld quality but lead to substantial increases in manufacturing difficulty and cost. Meanwhile, the interaction between the tool and workpiece becomes more complex, requiring more precise control of process parameters to avoid defect formation. To tackle these challenges, researchers continue to explore optimal design methods for welding tools. The welding tool serves as the core component of FSW technology, and its functional design directly affects temperature distribution, material flow, and microstructural evolution during welding. Such impacts ultimately determine the performance and quality of welded joints [98]. The fixed-shoulder tool developed by Eslami et al. [99] promotes material mixing and flow by maintaining constant welding force, reduces thermal stress concentration and deformation, and significantly improves weld-surface quality. Al-Sabur et al. [100] developed a pneumatic portable welding device focusing on defect repair in FSW. Although the joint performance is slightly inferior to that of traditional FSW, it optimizes weld surface morphology, providing new possibilities for the application of FSW in defect repair. In addition, the application of auxiliary methods such as induction heating, hot shoe technology, and external heating systems offers new directions for optimal tool design. Nath et al. [101] developed a self-heating FSW tool for PP welding. In traditional welding processes, increased welding speed is usually accompanied by reduced heat input, but the unique design of the self-heating tool effectively balances heat input, improves stir zone uniformity and material flow characteristics, reduces residual undeformed material at the joint, and ensures uniform material mixing; the macro-morphology of the joint cross-section is shown in Figure 11. Bagheri et al. [102] adopted “hot shoe” technology for ABS joining. The distinctive feature of the hot shoe tool is its shoe-shaped shoulder structure, which is stably mounted on bearings and remains stationary during tool rotation. Positioned directly above the weld and heated by an external heat source, the hot shoe fully covers the weld zone, prevents molten plastic overflow, and improves the smoothness of the welding zone.

Friction stir welding, as a solid-state joining technology featuring low welding temperature, small deformation, and environmental friendliness, has been extensively researched and applied in dissimilar material joining, particularly the joining of metals and polymers. Although the core research focus of this paper lies in the dissimilar joining of metals and polymers, researchers have also conducted in-depth studies on the FSW joining of various polymer materials. Such research on dissimilar polymers is not only an important part of FSW technology development but also provides valuable references for addressing key issues (e.g., material compatibility, heat distribution, and interface bonding) in the FSW joining of metals and polymers. To clarify the research progress in this field, it is necessary to systematically sort out the relevant studies on FSW joining of various polymer materials carried out by scholars. At present, FSW has realized the joining of various polymer materials. By designing welding tools with different geometric structures, the defects and strength of polymer FSW joints are summarized in Table 2, showing that the tensile properties of all welded joints are lower than those of the base materials. Panneerselvam et al. [103] successfully joined PA6 using a tool with a left-handed threaded pin and found that counterclockwise rotation of the tool produces defect-free welds with excellent joint performance. Liu et al. [104] demonstrated that a tool with a conical pin can enhance the fluidity of polymer melt in the welding zone and optimize welding effects. Studies by Derazkola et al. [105] and Mendes et al. [106] focused on the influence of pin surface area and shape on the welding process, revealing that pins with large surface area and relatively smooth surface can stir materials more effectively; maintain temperature uniformity during welding; and prevent the formation of defects, such as voids, in welds. Meanwhile, Mendes welded ABS using a tool with a fixed shoulder and external heating system. High axial force and external constant temperature heating system reduced the temperature gradient of welds, avoiding shrinkage and void defects. Although the heat-assisted fixed-shoulder FSW tool designed by Moochani et al. [107] enables efficient joining of PP, the use of a controllable hot air gun increases equipment complexity and cost. Similarly, the induction-heated tool studied by Vijendra et al. [108] can precisely regulate tool temperature, but the introduction of a feedback control system increases system complexity and maintenance difficulty. In addition, Banjare et al. [109] and Kumar et al. [110] adopted auxiliary heating tools and double-step square-shoulder tools for polymer joining, respectively; such tool designs solve the problems of material loss and uneven heat distribution during welding. Studies by Panneerselvam et al. [111] and Payganeh et al. [112] further discussed the influence of pin morphology on the welding process and joint performance; pins of different shapes generate different heat input and shear force during stirring, affecting material flow and bonding effects.

In summary, the selection and optimal design of tool geometric structure play a crucial role in the FSW of polymer-matrix materials and metals. From early research exploration to current innovative design, the efforts of scholars have not only solved many problems of traditional tools but also developed a series of tools with excellent performance.

### 3.3. Effects of Welding Parameters on Defect Formation in Joints

**Figure 12:**
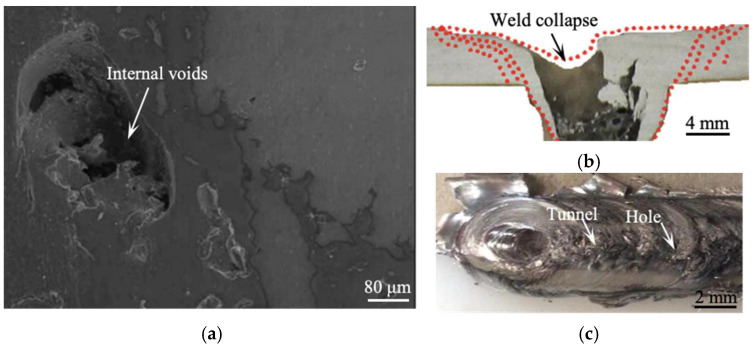
General diagram of the influence of process parameters on joint defects. (**a**) Hole defect in the joint [119]. (**b**) Weld collapse defect [114]. (**c**) Tunnel flash defect on weld surface [120].

Due to the considerable differences in physical and chemical properties between polymer-matrix materials and metals, the internal and external regions of the joints are prone to defects such as voids, tunnels, and undercuts under the influence of varying heat input, as illustrated in Figure 12, which further weakens the joint strength. Extensive studies have been conducted to comprehensively investigate weld quality by carefully adjusting key process parameters, including tool rotational speed, welding speed, dwell time, and tilt angle [1]. Shahmiri et al. [113] and Derazkola et al. [114] successfully regulated the heat distribution in the stir zone by varying the rotational and welding speeds, which increased the thickness of the interdiffusion layer and effectively suppressed defect formation. Ratanathavorn et al. [115] observed that excessively high welding speed resulted in larger metal fragments and the formation of void defects at the dissimilar material interface. Furthermore, Patel et al. [116] investigated the effect of the ratio of welding speed to rotational speed on the joint strength between 6061 aluminum alloy and PC, revealing that a low speed ratio led to insufficient material bonding, whereas a high speed ratio caused excessive polymer melting and increased joint defects. Malaske et al. [117] also pointed out that increasing the rotational speed or decreasing the welding speed promoted excessive melting of the polymer and degraded the joint performance. Pereira et al. [118] joined 6082 aluminum alloy and PA6 by varying the plunge depth; increasing the plunge depth did not significantly enlarge the bonded area but drove more aluminum into the polymer matrix. Rahmat et al. [119] evaluated the surface morphology of 7075 aluminum alloy and PC joints, and found that valley-shaped defects readily formed on the weld surface when the rotational speed and welding speed were improperly matched. Similarly, Khodabakhshi et al. [120] reported that excessive heat input led to material degradation and tunnel defects during the FSW of 5059 aluminum alloy and HDPE. Wang et al. [121] performed butt welding experiments on LC4 aluminum alloy and polytetrafluoroethylene (PTFE) sheets, and found that an overly high rotational speed softened and warped the PTFE material, while a tool tilt angle of 2° yielded the best welding performance. Pereira et al. [122] studied the influence of dwell time on the welding results of 6082-T6 aluminum alloy and PA6. Although porosity defects increased with longer dwell time, the enlarged bonded area improved the tensile strength. Ogawa et al. [123] explored the effect of welding time on the fatigue strength of 5182 aluminum alloy/CFRP joints; the melting degree of the polymer increased with welding time, leading to enhanced fatigue strength of the joints.

The effects of process parameter adjustment on the mechanical properties of welded joints are summarized in Table 3. The influence of individually varying welding parameters on joint strength often exhibits significant variations depending on the materials employed. Studies by Fan et al. [124], Kordestani et al. [125], and Kumar et al. [126] highlighted that tool tilt angle and rotational speed are critical to welding quality. A proper tilt angle facilitates vertical material flow and favors pore elimination, whereas an excessive tilt angle may induce the escape of molten material and the formation of flash defects, as reported by Ahmadi et al. [127]. Using the Taguchi method, Bozkurt et al. [128] demonstrated that tool rotational speed presents the most significant effect among welding parameters, with a contribution ratio as high as 73.85%. Meanwhile, Nagatsuka et al. [129] observed that decreasing welding speed remarkably prolongs the duration of melting and thermal decomposition of CFRP, thus impairing the welding quality. Bilici et al. [130] identified pre-weld dwell time as the dominant factor governing joint strength, followed by tool rotational speed. Finally, Dashatan et al. [131] emphasized that an insufficient post-weld dwell time allows molten polymer to flow out of the weld zone, preventing the formation of sound joints.

### 3.4. Research Status of Optimized Friction Stir Welding Processes for Polymer-Matrix Materials and Metals

In recent years, dissimilar joining between polymer-matrix materials and lightweight alloys, especially via friction stir welding, has attracted extensive attention. Research in this field has been mainly focused on improving the mechanical properties of welded joints [132]. To this end, various optimization strategies have been adopted, including surface modification, structural design of auxiliary features, and particle reinforcement.

**Figure 13:**
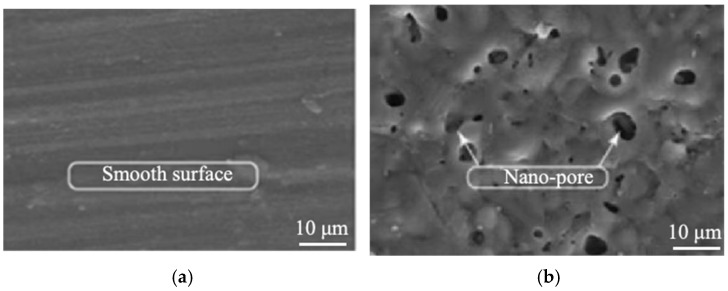
Surface morphology of the aluminum alloy. (**a**) Untreated surface [135]. (**b**) PEO-treated surface [135].

Surface modification techniques form a modified layer on the sheet surface through chemical or physical treatments, which improve wettability and interfacial compatibility, promote interfacial reactions, and enhance the mechanical performance of joints. Liu et al. [133] applied plasma electrolytic oxidation (PEO) on magnesium alloy and corona discharge treatment on polyethylene (PE). It was found that the porous structure generated on the PEO-treated magnesium alloy surface accommodated molten PE and strengthened the micromechanical interlocking between the two materials, thus achieving high-strength bonding. On the other hand, Goushegir et al. [134] reported that the shear strength of joints between 2024 aluminum alloy and CFRP was nearly 10 times higher after phosphoric acid anodizing and primer pretreatment compared with untreated joints. Furthermore, Meng et al. [135] also implemented PEO treatment on aluminum alloy, producing a nanoporous structure, as illustrated in Figure 13. Such nanopores improved the permeability between the metal and molten CFRP, further enhancing micromechanical interlocking.

**Figure 14:**
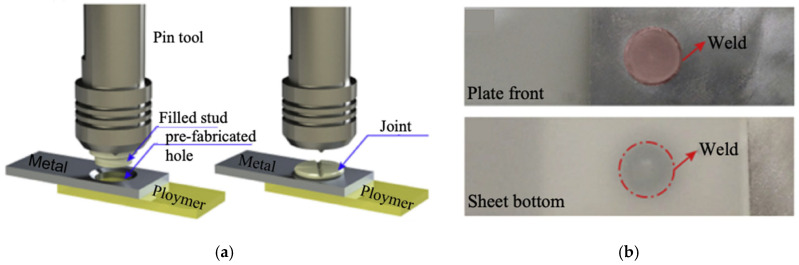
Schematic diagram of physical filling welding and welding results. (**a**) Schematic diagram of stud-filling welding [141]. (**b**) Post-welding morphology [141].

Auxiliary structural manufacturing optimizes the workpiece before welding, including laser texturing to modify surface characteristics and pre-drilling with inserted studs. Han et al. [136] fabricated deep-hole structures on aluminum alloy via laser pretreatment, which promoted the formation of Al-O-C chemical bonds between aluminum oxides, thermal oxidation products, and PP, thereby strengthening interfacial bonding. Jiang et al. [137] treated aluminum alloy surfaces using laser texturing and achieved high-quality joining with PEEK. Microstructural analysis revealed both mechanical interlocking and chemical bonding, with the formation of Al-O-C bonds contributing to improved joint strength. Bi et al. [138] created bionic textures on 6061-T6 aluminum alloy using femtosecond laser to produce high-strength hybrid structures with CFRP. The dual-scale roughness induced by femtosecond laser treatment promoted carbon incorporation into alumina and enhanced covalent Al-O bonding, thus increasing interfacial strength. Wu et al. [139] also employed laser pretreatment on 5052 aluminum alloy to create a rough porous surface, enabling PA6 to infiltrate the porous structure and enlarge the interfacial bonding area. Gao et al. [140] joined 7075 aluminum alloy to polyimide (PI) sheets; laser texturing on aluminum alloy increased tensile–shear load, although no chemical bonding was detected at the interface. Liu et al. [6] achieved high-strength joints between aluminum alloy and CFRP by combining friction stir lap welding with laser surface texturing. By optimizing texturing parameters, softened PA66 fully infiltrated the textured grooves, greatly reducing interfacial voids and cracks in Al/CFRP joints. In addition, Huang et al. [141] proposed an innovative method involving pre-drilled holes on 6082-T6 aluminum alloy with additional stud filling. As shown in Figure 14, macro-mechanical bonding was first established between the metal and the stud. As the tool’s front edge contacted the hole edge, molten PP beneath the tool was extruded into the pre-drilled holes, forming a micro-bonding layer with plasticized metal. Chen et al. [142] investigated FSW joining between porous TC4 titanium alloy and PE, where PE successfully infiltrated the porous titanium structure, significantly improving shear strength.

**Figure 15:**
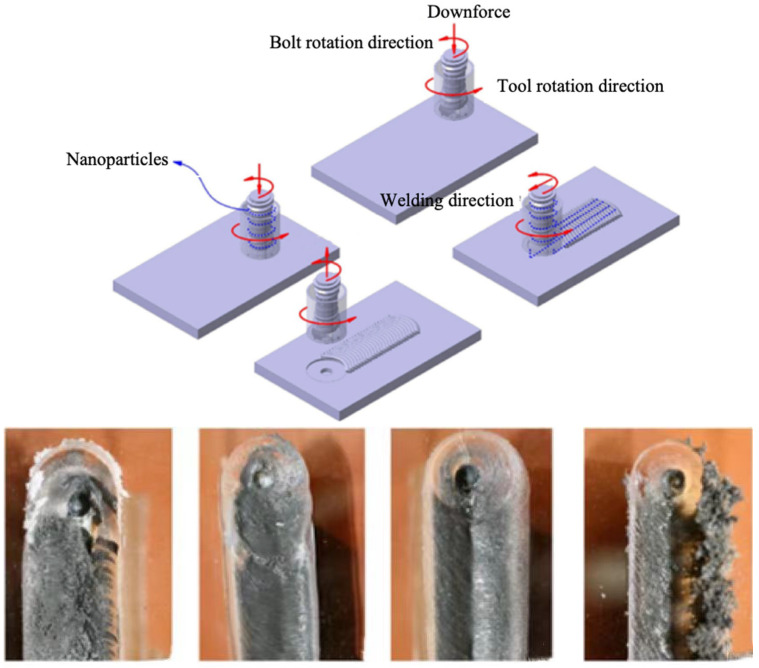
Schematic diagram of nano-filled FSW [144].

Particle doping introduces functional particles with high thermal conductivity and mechanical strength, such as carbon nanotubes or ceramic particles, into the polymer matrix. These particles participate in interfacial reactions during welding and remarkably improve bonding quality [143]. Derazkola et al. [144] injected alumina nanopaste into the stir zone during FSW of 2024 aluminum alloy and PC, as schematically illustrated in Figure 15, which effectively compensated for the property mismatch between aluminum and polymer. The nanoparticles enhanced interfacial flow, thickened the interaction layer, and strengthened the interface. MirHashemi et al. [49] studied joining between 7075 aluminum alloy and LDPE, and found that SiC nanoparticles incorporated in LDPE suppressed turbulence, inhomogeneous deformation, and poor mixing during dissimilar joining. Moreover, particle doping promoted the formation of fine aluminum fragments with refined equiaxed grains inside the polymer, reinforcing mechanical interlocking. Singh et al. [145] focused on the joint interface between LDPE and HDPE using iron powder as filler. Upon melting, polymer coalescence occurred, and iron powder established robust bonding between the joint and the metal. Andre et al. [146] introduced an additional PPS film as an interlayer during friction spot joining of 2024-T3 aluminum alloy and CFRP. This design expanded the plastic deformation and adhesion zones, efficiently filling micro-gaps at the interface. Ozlati et al. [147] embedded PP filaments into lap joints between PP and Al-Mg alloy sheets. Joints exhibited excellent ductility when the filaments were aligned parallel to the tensile loading direction.

**Figure 16:**
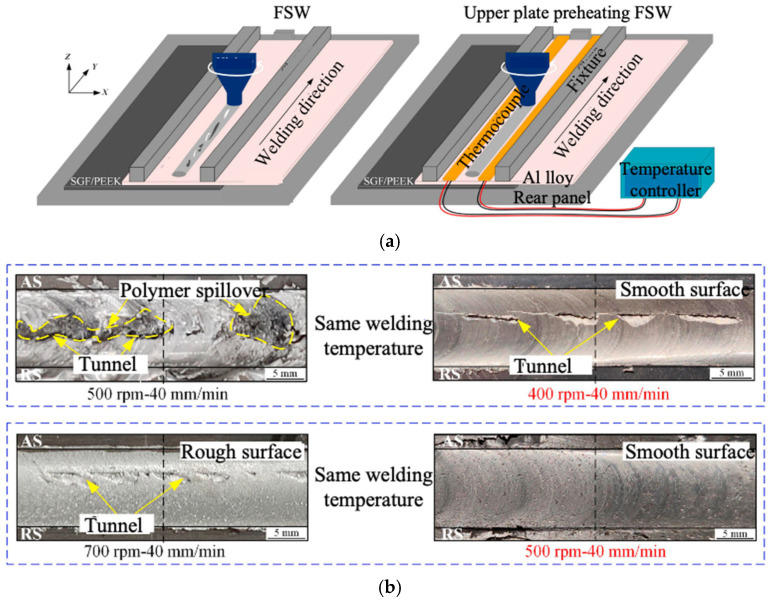
Schematic diagram of preheating welding and welding results. (**a**) Schematic diagram of upper plate auxiliary heating FSW [148]. (**b**) Surface morphology of weld before and after auxiliary heating [148].

Recently, several researchers have explored external field-assisted FSW based on optimized material flow. Li et al. [148] proposed a heating-assisted FSW technique for lap joints. Welding experiments between 7075 aluminum alloy and SGF/PEEK verified that auxiliary heating regulated polymer flow and reduced overflow onto the weld surface, as shown in Figure 16. Aydin et al. [149] preheated PE near its melting point to ensure uniform heat distribution, promote material mixing, and reduce the risk of over-melting. Banjare et al. [109] developed a heat-assisted tool for thermoplastic FSW which significantly reduced chip formation and material loss while improving surface smoothness. Joo et al. [150] investigated the effect of external heating on magnesium alloy FSW joints and observed defect-free welds compared with conventional processes. Raju et al. [151] studied FSW of thermoplastics, including nylon and HDPE, using induction-assisted heating coils; higher temperatures and lower traverse speeds yielded the most desirable results. Sun et al. [152] performed heating-assisted FSW of 6061-T6 aluminum alloy and PC, showing that auxiliary heating remarkably enhanced mechanical properties compared with unheated joints. Furthermore, Nagarajan et al. [153] examined the influence of air- and water-cooling media on lap joints between PC and 5052-H32 aluminum alloy. Water cooling improved microstructural compactness and increased shear strength by 16%. Yaduwanshi et al. [154] demonstrated that water cooling eliminated unnecessary particle aggregation at grain boundaries and enhanced yield strength. Sun et al. [155] used the Taguchi method and ANOVA to analyze the effects of rotational speed, welding speed, and water temperature on the shear strength of underwater PC FSW joints, identifying welding speed as the most influential parameter. Sandeep et al. [156] compared air and water cooling during FSW of 7475 aluminum alloy and PPS, and reported that water cooling effectively suppressed void defects commonly observed in air environments.

In summary, extensive studies have been conducted on the FSW of neat polymers and metals under single-pass welding conditions, covering tool geometry design, process parameter optimization, and auxiliary technologies. These efforts have significantly improved the formability and mechanical performance of polymer–metal FSW joints, providing valuable references for understanding the structure–property relationships of dissimilar polymer-matrix composite/metal joints. However, several critical challenges remain: The bonding interface between CFRP and aluminum alloy produced by FSW often exhibits poor formation quality, high surface roughness, low yield, and difficulty in achieving high-performance joints. Meanwhile, the large discrepancy in thermophysical properties between dissimilar materials makes CFRP susceptible to fiber degradation and matrix resin decomposition at elevated temperatures, leading to porosity and other defects that weaken interfacial bonding strength. Therefore, key issues regarding bonding mechanisms, defect control, and property optimization of CFRP–aluminum alloy joints still require further investigation.

## 4. Conclusions

(1) Comparative analysis of joining processes reveals that laser welding, induction welding, resistance welding, and ultrasonic welding each present distinct limitations in CFRP–metal joining, primarily related to thermal degradation, uneven heating, inconsistent weld quality, or geometric constraints. Friction stir welding (FSW) offers the most balanced performance, combining single-pass long-distance capability, low environmental impact, and superior industrial applicability, contingent upon effective thermal management and interface optimization.
(2) The optimal FSW processing window for CFRP–aluminum alloy joints is a tool rotation speed of 1200–1500 rpm with a traverse speed of 30–50 mm/min, maintaining the CFRP side below its thermal degradation threshold (350 °C) and achieving a maximum joint tensile strength of 78 MPa (65% of base CFRP strength).
(3) Interfacial bonding relies on dual mechanisms of mechanical interlocking and localized chemical bonding, though effective bonding covers only approximately 30% of the interfacial area, remaining the primary constraint on joint performance.
(4) Surface modification, auxiliary structures, nanoparticle reinforcement, and external field assistance effectively enhance joint quality by improving wettability and suppressing defects.
(5) Future research should focus on multi-physics coupled simulation, intelligent parameter optimization, and specialized tool development to enable reliable engineering application of CFRP–aluminum alloy FSW technology.

## Figures and Tables

**Table 1:** Summary table of the literature on material connection mode and joint structure.

Material	Bonding Method	Joint Bonding	Cited
1120 Al alloy and HDPE	FSW	Melted polyethylene flows into aluminum and its oxides	[60]
6061-T6 Al alloy and CFRP		Aluminum anchor permeates into composite material	[61]
5058 Al alloy and PMMA		Semi-sharp “U-shaped antler” structure bonding interface	[62]
5052 Al alloy and PP		Aluminum hooks reinforce the joint area composed of aluminum scrap and PP	[63]
6014 Al alloy and CFRP		The polymer–metal melt layer is the adhesive layer of the material	[65]
1050 Al alloy and CFRP		Surface undulation of aluminum alloy deformed by CFRP embedding	[66]
6082-T6 Al alloy and ABS		The joint has a staggered structure	[69]
Cu and PMMA	FSSW	The polymer material is pushed into the rough metal surface and accumulated along the bending area of the copper sheet	[72]
Cu and CFRP	FLJ	A thin Cu_2_O transition layer is formed at the interface	[73]
AZ31 Mg and CFRP	FSI	After being softened by heating, CFRP flows into the gaps between magnesium alloy base materials and interlocking structures formed by magnesium alloys	[75]

**Table 2:** Defects and strength of polymer FSW joint.

Material	Pin Tool Geometry	Defect	Shear Strength (MPa)	Cite
PA6	Left-hand thread stirring pin	Pores and cavities	34.8	[103]
PE	Cylindrical and conical stirring pin	Pore	36.4	[104]
PMMA	Conical and triangular stirring pin	Tunnels and voids	43.0	[105]
ABS	Long fixed shoulder	Pores and voids	31.2	[106]
PP	Thermally assisted fixed shoulder	Crack	The tensile strength of welded joint can reach 96%	[107]
HDPE	Induction heating pin tool	Flash	28.3	[108]
PP	Auxiliary heating pin tool	Voids, lack fusion	14.6	[109]
ABS/PC	Double shoulder pin tool	Tunnels and cracks	23.1	[110]

**Table 3:** Optimization process parameters of polymer FSW joint strength.

Material	Welding Process Parameters	Strength (MPa)	Cite
PP	The inclination angle is 3°, the rotation speed is 730 r/min, and the welding speed is 20 mm/min.	66.0	[124]
PP	The inclination angle is 6°, the rotation speed is 2000 r/min, and the welding speed is 8 mm/min.	10.7	[125]
PP	The inclination angle is 1°, the rotation speed is 1250 r/min, and the welding speed is 25 mm/min.	42.2	[127]
HDPE	The inclination angle is 3°, the rotation speed is 3000 r/min, and the welding speed is 115 mm/min.	19.4	[128]
HDPE	The residence time is 60 s, and the rotating speed is 700 r/min.	33.3	[130]
PMMA/ABS	The residence time is 30 s, the rotating speed is 800 r/min, and the welding speed is 8 mm/min.	13.5	[131]

## Data Availability

The original contributions presented in this study are included in the article. Further inquiries can be directed to the corresponding author.
